# An experimental study of levamisole incorporated diet on fish health and resistance against* Pseudomonas aeruginosa* isolated from* Oreochromas niloticus*

**DOI:** 10.1038/s41598-025-96914-7

**Published:** 2025-04-26

**Authors:** Ghada A. El-Gammal, Adel M. El-Gamal, M. A. Rashed, Amina S. Kassab, Aly S. Saif, Sabreen E. Fadl

**Affiliations:** 1 Kafrelsheikh Lab, Bacteriology Unit, Agricultural Research Center (ARC), Animal Health Research Institute, Giza, Egypt; 2 Kafrelsheikh Regional Lab, Fish Diseases Unit, Agriculture Research Center (ARC), Animal Health Research Institute, Giza, Egypt; 3 Kafrelsheikh Provincial Lab, Biochemistry, Nutritional Deficiency Diseases and Toxicology Unit, Agricultural Research Center (ARC), Animal Health Research Institute, Giza, Egypt; 4 Biochemistry Department, Faculty of Veterinary Medicine, Matrouh University, Matrouh, Egypt

**Keywords:** *Pseudomonas aeruginosa*, Nile tilapia, Growth performance, Immunity, Levamisole, Biological techniques, Immunology

## Abstract

*Pseudomonas aeruginosa* (*P. aeruginosa*) is one of the most common ones that harm fish. *P. aeruginosa* has been regarded as one of the most significant threats to the fishing industry, which also affects public health. Thus, the present investigation was done in two steps; the first step was to examine the prevalence and the antibiogram of *P. aeruginosa* among Nile tilapia (*Oreochromas niloticus *(*O. niloticus*)) from aquaculture farms in Kafr El-shiekh Governorate with an emphasis on their antibiotic resistance genes (*BlaTEM, tetA,* and *sul1*). The second step was to investigate the effect of levamisole as a feed supplement for tilapia fish on growth performance, immunity, serum biochemistry, and the protective effect against artificial infection with the previously isolated in the first step *P. aeruginosa* strain. One hundred samples were collected from morbid Nile tilapia fish in the first step. The incidence of *P. aeruginosa* was 14%. Susceptibility of *P. aeruginosa* isolates to 9 antimicrobial agents showed that about half of *P. aeruginosa* isolates were multidrug-resistant (MDR) to (5–6) antibiotics. All of the isolates were sensitive to amikacin, ciprofloxacin, and norfloxacin (100%), and half of them were resistant to azithromycin, amoxicillin with clavulanic, tetracycline, and sulfa with trimethoprim. *P. aeruginosa* isolates were confirmed diagnosed using the 16S rRNA gene, which was detected in 100% of the tested isolates, and was also evaluated for the presence of antibiotic resistance genes (*blaTEM, tetA,* and *sul1*), which were 85.7%, 85.7%, and 100%, respectively. In the second step, a 2-month feeding trial was performed on 160 *O. niloticus* fish with a weight of 56.75 ± 3 g. Fish were randomly distributed into four groups, each at a rate of 10 fish per aquarium in four replicates, and fed on a diet containing 0.0, 500, 750, and 1000 mg levamisole/kg diet. At the end of the feeding trial, fish were challenged by pathogenic *P. aeruginosa,* which was isolated in the first step. The results of the in vivo trial showed that levamisole safely improved the growth and immunity of Nile tilapia without side effects on liver function.

## Introduction

Aquaculture of tilapia is a rapidly expanding sector that provides humans with a source of animal protein. Egypt’s Nile tilapia aquaculture produced a substantial volume, accounting for roughly 13.8% of all fish raised for food worldwide^1^. A normal component of fish microbiota, *Pseudomonas aeruginosa *(*P. aeruginosa*) can become extremely pathogenic under stressful circumstances, leading to serious illnesses such as hemorrhagic septicemia, lethargy, decreased appetite, gill necrosis, abdominal distension, splenomegaly, friable liver, red spots on the skin, or ragged fins^2^. Additionally, Ndi and Barton^3^ and Shahrokhi et al.^4^ reported that the bacterium *P. aeruginosa* is one of the most common ones that harm fish. *P. aeruginosa* has been regarded as one of the most significant threats to the fishing industry, which also affects public health^5^. Often, the diseased fish will keep the fins close to its body and may open and close its mouth rapidly. However, the infection will kill the fish before obvious symptoms develop^6^. To get past the host’s immune system, virulent bacteria release poisons and enzymes that break down tissue. Extracellular products and cell surface structures that serve as adhesion factors or in other ways throughout the infection process^7^. Furthermore, *P. aeruginosa* contains both cell-mediated and secreted virulence determinants. The cell-mediated virulence factors, including lipopolysaccharide (LPS), flagella, and pili, play a vital role in motility, colonization of bacteria in the host tissues, and the invasion of bacterial active proteins into the target cells^8^. Besides, the secreted virulence types enable microbial invasion and propagation, strengthen inflammation, resulting in damage to host tissue, and increase infection severity. The most common secreted virulence determinants accompanying *P. aeruginosa* are exotoxin A and exotoxin S. Exotoxin A is accountable for the prevention of protein synthesis in the host cell, whereas exotoxin S is an extracellular protein that is implicated in cell-apoptosis through the initiation of the GTPase and ribosyltransferases actions^9^. Important constraints in the aquaculture sector include immunosuppression, increased prevalence of infectious diseases, and decreased fish survival as a result of stress^10^. Additionally, the rise of antibiotic-resistant bacteria, changes in the intestinal microbiota of fish, antibiotic residues in fish meat, and worries about food security have put pressure on aquaculture to use fewer antibiotics and chemical medicines^11^.

Due to the serious health issues, it creates for both humans and animals, *P. aeruginosa* is ranked among the top ten most hazardous malignant strains worldwide^12^. Multidrug-resistant (MDR) *P. aeruginosa* is frequently linked to nosocomial infections and is becoming a significant hazard in relation to poor patient outcomes^13^. The Centre’s for Disease Control and Prevention (CDC) now considers *P. aeruginosa* to be a significant bacterium, and the World Health Organization (WHO) placed it in the category of highest priority in their 2017 global priority list of pathogens^14^.

Multidrug resistance has been increased all over the world, which is considered a public health threat. Several recent investigations reported the emergence of multidrug-resistant bacterial pathogens from different origins that increase the necessity for the proper use of antibiotics. Besides, the routine application of antimicrobial susceptibility testing to detect the antibiotic of choice as well as the screening of the emerging MDR strains^15^. The major mechanisms of *P. aeruginosa* used to avoid antibiotic attack can be classified into intrinsic, acquired, and adaptive resistance. The intrinsic resistance includes low outer membrane permeability, expression of efflux pumps that expel antibiotics out of the cell, and the production of antibiotic-inactivating enzymes such as β-lactamases. The acquired resistance can be achieved by either mutational changes or the acquisition of resistance genes via horizontal gene transfer^16^. Adaptive resistance through transient alterations in gene and/or protein expression in response to an environmental stimulus, and it is reversible when the stimulus is removed^17^. In *P. aeruginosa*, the best-characterized mechanisms of adaptive resistance are the formation of biofilm and the generation of persister cells, which result in persistent infection and poor prognosis. The impact of antimicrobial resistance includes treatment failures, resulting in economic losses, and the potential to serve as a reservoir of resistant bacteria. Therefore, the selection of appropriate antimicrobial agents should be a top priority^18^. Also, molecular typing of the majority of inherited antibiotic-resistance genes should be carried out.

Compared to conventional antibiotic treatments, the use of dietary immunostimulants, such as compounds of synthetic origin, is thought to be a preventive strategy for fish diseases^19^. Levamisole hydrochloride is one such instance; it is a synthetic imidazothiazole derivative anthelmintic that is used to treat parasite diseases in fish, cattle, and humans^20^. When included in fish diets, such as Nile tilapia^21^, it has been shown to positively affect the immune system^22^. Moreover, de Azevedo et al.^23^ reported that feeding levamisole to fish provided the best benefits to Nile tilapia. However, Levamisole has immunostimulatory effects when taken orally and can be delivered by injection or immersion^24^.

Overall, one potential aquaculture tactic is the use of levamisole to enhance fish welfare and stop disease outbreaks. Thus, this study was done in two steps, the first step was to examine the prevalence and the antibiogram of *P. aeruginosa* among Nile tilapia from aquaculture farms in Kafr El-shiekh Governorate with an emphasis on their antibiotic resistance genes (*blaTEM, tetA,* and *sul1*). The second step was to investigate the effect of different doses of levamisole as a feed supplement for tilapia fish on growth performance, immunity, serum biochemistry, and the protective effect against artificial infection with the previously isolated in the first step *P. aeruginosa* strain.

## Materials and methods

### First step: prevalence and the antibiogram of *P. aeruginosa* among Nile tilapia

#### Samples of morbid Nile tilapia fish collection

A total of 100 Nile tilapia fish samples were randomly collected from different fish farms at Kafr El-shiekh Governorate, Egypt. After that, the gathered samples were put in plastic bags containing farm water aerated with oxygen, and transported to the microbiology unit, Animal Health Research Institute, Kafr El-shiekh Lab. and immediately analyzed for clinical signs and gross lesions of all diseased fish in accordance with Austin and Austin^25^.

#### *Pseudomonas aeruginosa* isolation and identification

After being cultivated on Tryptic soy broth (TSB, Hi-media India) for 24 h at 37 °C, one loopful of the inside organs that were gathered and pooled (liver, spleen, kidney, and heart) was streaked onto Cetrimide agar (Hi-media India) and MacConkey’s agar (Oxoid, UK) and incubated for 24 h at 37 °C under aerobic conditions. The usual *P. aeruginosa* isolates exhibited a yellowish-green fluorescent color and had huge irregular colonies with a fruity odor. Every probable colony was collected and refined for biochemical and phenotypic traits. All isolates were identified biochemically according to Algammal et al.^26^. Briefly positive for catalase, oxidase, nitrate reduction, citrate utilization, and gelatin liquefaction whereas negative for indole, methyl red, VP, urea hydrolysis, and H_2_S production, and visually using Gram’s stain showed typical Gram-negative, medium-sized, straight, motile, and non-sporulated bacilli. Additionally, a species-specific set of primers that target *16S rRNA* was used to confirm the isolates that were found.

#### Antimicrobial susceptibility

A disc diffusion method was used to assess the retrieved isolates’ susceptibility to various commercial antimicrobial agents purchased from Oxoid (UK); nine antimicrobials belonging to six classes of antibiotics were used, such as azithromycin (15 µg), amoxicillin with clavulanic acid (30 µg), doxycycline (30 µg), tetracycline (30 µg), ciprofloxacin (5 µg), norfloxacin (10 µg), gentamicin (10 µg), amikacin (30 µg), and sulfa trimethoprim (25 µg). Muller Hinton Agar plates (Oxoid, UK) were used for the test, and they were incubated for 24 h at 37 °C. Single colony was resuspended in 0.9% saline. The concentration was adjusted to McFarland 0.5 (1.5 × 10^8^ CFU/mL) according to the McFarland scale, evenly coated on Muller Hinton Agar (Oxoid, UK) using a sterile swab, and then discs of antibiotics were dispersed and incubated at 37 °C for 18–24 h. The test was carried out in compliance with the Clinical Laboratory Standards Institute’s guidelines^27^. By measuring the inhibitory zone widths, bacterial isolates were categorized as susceptible (S), intermediate (I), or resistant (R). *E. coli* ATCC25922 was taken as a reference strain.

Determination of MAR index in brief, the MAR index was calculated by using the formula MAR = a/b, where “a” refers to the number of antibiotics to which isolates demonstrated resistance and “b” accounts for all of the used antibiotics. A value of more than 0.2 suggests that the isolates are from high-risk sources^28^. Interpretation of antibiotic susceptibility to evaluate MDR, XDR, and PDR was identified according to Magiorakos et al.^29^.

#### Confirmation of the isolates and detection of some antibiotic resistance genes using PCR

##### DNA extraction

With some adjustments from the manufacturer’s instructions, the QIAamp DNA Mini kit (Qiagen, Germany, GmbH) was used to extract DNA from isolates. In short, 200 µl of the sample suspension was treated for 10 min at 56 °C with 200 µl of lysis buffer and 10 µl of proteinase K. Following incubation, the lysate was mixed with 200 µl of 100% ethanol. After that, the sample was cleaned and centrifuged following the manufacturer’s instructions. 100 µl of the elution buffer included in the kit was used to elute the nucleic acid.

##### Oligonucleotide primer

Primers used were supplied from **Metabion (Germany**) are listed in Table 1.

**Table 1:** Primers sequences, target genes, amplicon sizes, and cycling conditions.

Target gene	Primers sequences	Amplified segment (bp)	Primary denaturation	Amplification (35 cycles)			Final extension	References
				Secondary denaturation	Annealing	Extension		
*blaTEM*	ATCAGCAATAAACCAGC	516	94 °C 5 min	94 °C 30 s	54 °C 40 s	72 °C 45 s	72 °C 10 min	Colom et al.^30^
	CCCCGAAGAACGTTTTC							
*Sul1*	CGG CGT GGG CTA CCT GAA CG	433	94 °C 5 min	94 °C 30 s	50 °C 40 s	72 °C 45 s	72 °C 10 min	Ibekwe et al.^31^
	GCC GAT CGC GTG AAG TTC CG							
*tetA*(*A*)	GGTTCACTCGAACGACGTCA	570	94 °C 5 min	94 °C 30 s	50 °C 40 s	72 °C 45 s	72 °C 10 min	Randall et al.^32^
	CTGTCCGACAAGTTGCATGA							
*P. aeruginosa 16S Rdna*	GGGGGATCTTCGGACCTCA	956	94 °C 5 min	94 °C 30 s	52 °C 40 s	72 °C 1 min	72 °C 10 min	Spilker et al.^33^
	TCCTTAGAGTGCCCACCCG							

##### PCR amplification

Primers were used in a 25 µl reaction that included 6 µl of DNA template, 4.5 µl of water, 12.5 µl of EmeraldAmp Max PCR Master Mix (Takara, Japan), and 1 µl of each primer at a concentration of 20 pmol. An Applied Biosystem 2720 heat cycler was used to carry out the process.

##### Analysis of the PCR products

Using gradients of 5 V/cm, the PCR products were separated by electrophoresis on a 1.5% agarose gel (AppliChem, Germany, GmbH) in 1 × TBE buffer at room temperature. Twenty microliters of the products were put into each gel slot for the gel analysis. Fragment sizes were measured using a generuler 100 bp ladder (Fermentas, Thermo, Germany). A gel documentation system (Alpha Innotech, Biometra) took pictures of the gel, and computer software was used to analyze the data.

### Second step: in vivo experiment

#### Experimental design

160 fish (56.75 ± 3 g/fish at an age of about 220 days) were collected from a private fish farm in Kafrelsheikh, Egypt. After a 2-week acclimatizing period, healthy fish were randomly divided into four groups in glass aquariums (each group has 4 replicates, n = 40 fish/group, 10 fish/replicate) and excluded all skewed results in static. G1 (control group) was provided with the basal diet (Table 2)^34^ without any additives. G2 (500 mg levamisole/kg ration). G3 (750 mg levamisole/kg ration) and G4 (1000 mg levamisole/kg ration). Levamisole Hydrochloride is a synthetic imidazothiazole derivative that was obtained from Sigma Chemical Company (USA). Throughout the trial, daily partial replacements of dechlorinated water were made to the aquarium’s water, with adjustments made to temperature, salinity, pH, and oxygen content at 24 °C, 1.1–2‰, 7.4–8.1, and 5.8–6.1 ppm, respectively. Moreover, throughout the feeding trial (8 weeks), all fish groups received feedings twice a day at 9 AM and 3 PM, at a rate of 3% of their body weight. Every 2 weeks, fish were weighed and counted for preparation of the diet. All growth parameters were computed, including feed conversion ratio (FCR), body weight (BW), and weight gain (WG). All experimental procedures were carried out according to the National Institutes of Health (NIH) general guidelines for the care and use of laboratory animals. The Institutional Animal Care and Use Committee of Animal Health Research Institute, Giza, Egypt, has approved the experimental design and procedures used in the study (83,429). All methods were carried out in accordance with relevant guidelines and regulations. The authors confirm that the study was carried out in compliance with the ARRIVE guidelines.

**Table 2:** Chemical composition and analysis of the basal diet.

Ingredients%	Chemical composition	Chemical analysis	
Yellow corn (7.6%)	327.5	Moisture%	6.8
Corn gluten (56.6%)	110	Dry matter%	93.2
Fish meal (47.8)	100	Crude protein%	30.3
Soybean meal (41.4%)	400	Ether extract%	6.4
Wheat bran (14.2%)	20	Ash%	2.9
Soya oil	35	Crude fiber%	4.2
Salt	2.5	NFE%	49.38
Mineral-vitamin premix*	3	Calcium%	0.82
Carboxy methyl- cellulose	2	Total phosphorus%	0.695
		Lysine%	1.76
Total	1000	Methionine%	0.66
		DE**	3146.9

*Mineral and Vitamins mixture- each one kg contains: vitamin D3 2,200,000 IU, vitamin A 12,000,000 IU, vitamin K3 2 g, vitamin C 250 g, vitamin E 10 g, vitamin B1 1 g, vitamin B6 1.5 g, vitamin B2 5 g, vitamin B12 0.01 g, Niacin 30 g, Folic acid 1 g, Biotin 0.050 g, and Pantothenic acid 10 g and carrier to 1000 g, Copper 4 g, Zinc 50 g, Iron 5 g, Manganese 60 g, Iodine 1 g, Selenium 0.1 g, Cobalt 0.1 g, calcium carbonate (CaCO3) carrier to 1000 g.

**Based on the chemical component of the utilized feedstuffs, digestible energy (DE) was computed (kcal/kg).

Before sampling, fish were anesthetized with 25 mg/L of tricaine methane sulphonate (MS-222). Blood samples from each group were taken at the end of the trial to assess the hemogram and leukogram. The experimental fish’s health was assessed using the serum samples. Aminotransferases (AST and ALT) activities were measured, as well as the amounts of total protein and albumin using serum samples that had been separated at 3000 rpm for 15 min using Bio-Diagnostic Company kits.

The investigators were not blinded during data collection. Blinding was used during analysis. Computational analysis was not performed blinded.

#### *Pseudomonas aeruginosa* experimental infection

The experimental challenge was performed with the isolated *P. aeruginosa* after sample collection. Fish were injected intraperitoneally with 1.5 × 10^8^ cells/ml (0.1 ml) from isolated bacteria^35^. After injection, fish were observed for 2 weeks for clinical signs and PM^36^.

### Statistical analysis

The minimum sample size required for a research study was ascertained by power analysis prior to the experiment’s commencement. Additionally, in order to demonstrate homoscedasticity and normality, the data were subjected to the Shapiro–Wilk and Levene tests for normal distribution. Then, statistical analysis of the acquired numerical data was performed using SPSS version 20 and one-way analysis of variance.

## Results

The results of this study were based on two main steps: isolating the *P. aeruginosa* from fish from Kafr El-Sheikh fish farms was the first step. Additionally, to know the incidence, antimicrobial susceptibility, and prevalence of this microbe in fish farms. The second step was feeding healthy tilapia fish on Levamisole for 2 months. At the end of the experiment, we used the microbe isolated from the first step to infect the fish and determined the effect of levamisole used to increase the resistance of fish to this experimental infection.

### First step: *P. aeruginosa* isolation

#### Clinical and P/M examination of naturally infected fish

Clinical examination of the collected fish samples reveals hemorrhage all over the skin, redness at the base of the fins with fin erosion, abdominal distension, detached scales, and eye opacity. Internally, the infected fish showed a gill lesion, an enlarged abdomen and spleen, and a pale liver.

#### Phenotypic characteristics of the recovered *Pseudomonas aeruginosa* isolates:

All the recovered *P. aeruginosa* isolates displayed large greenish colonies on the cetrimide agar (C. A.) medium. On MacConkey’s agar, it showed fat, smooth, non-lactose fermenting colonies with regular edges. Microscopic examination of these colonies showed typical Gram-negative, medium-sized, straight, motile, and non-sporulated bacilli. The biochemical characterization of these isolates was positive for oxidase, catalase, reduction of nitrate, citrate utilization, and gelatin hydrolysis, whereas they react negatively to indole, methyl red, VP, urea hydrolysis, and H_2_S production.

#### Incidence of *P. aeruginosa* among the examined Nile tilapia

A total of 14 *P. aeruginosa* isolates were recovered from 100 Nile tilapia with an isolation rate14%.

#### Antimicrobial susceptibility of ***Pseudomonas aeruginosa*** isolates (no = 14) Tables 3 and 4

**Table 3:** Antimicrobial susceptibility of *P. aeruginosa* isolates (no = 14).

Antimicrobial family	Antimicrobial agent	Sensitive		Intermediate		Resistant	
		No	%	No	%	No	%
Tetracyclines	Doxycycline (DO)	8	57.1	–	–	6	42.9
	Tetracycline (TE)	7	50	–	–	7	50
Aminoglycoside	Gentamicin (CN)	10	71.4	–	–	4	28.6
	Amikacin (AK)	14	100	–	–	–	–
Macroloid	Azithromycin (AZM)	7	50	–	–	7	50
β-Lactam-β-lactamase-inhibitor combination	Amoxicillin + Clavulanic (AMC)	4	28.6	3	21.4	7	50
Quinolone	Norfloxacin (NOR)	14	100	–	–	–	–
	Ciprofloxacin (CIP)	14	100	–	–	–	–
Sulphonamides	Sulfa\Trimethoprim (COT)	7	50	–	–	7	50

**Table 4:** Antimicrobial resistance patterns of *P. aeruginosa* isolates (n = 14).

No of isolates	Resistance pattern	MAR index	Resistance pattern
3 isolates	DO, TE, CN, AZM, AMC, COT	0.66	XDR
2 isolates	DO, TE, AZM, AMC, COT	0.55	XDR
1 isolate	DO, TE, CN, AZM, COT	0.55	MRD
1 isolate	TE, AZM, AMC, COT	0.44	MRD
1 isolate	AMC	0.11	–
6 isolates	No resistance	0	–

*DO* Doxycycline, *TE* Tetracycline, *CN* Gentamicin, *AZM* Azithromycin, *AMC* Amoxicillin + Clavulanic, *COT* Sulfa\Trimethoprim, *XDR* Extensive drug resistance, *MDR* Multi drug resistance.

The current investigation investigated the susceptibility of all *P. aeruginosa* isolates (14 isolates) to 9 antimicrobial agents and showed that about half of the *P. aeruginosa* isolates were multidrug-resistant (MDR) to (5–6) antibiotics. All of the isolates were sensitive to amikacin, ciprofloxacin, and norfloxacin (100%), and half of them were resistant to azithromycin, amoxicillin with clavulanic, tetracycline, and sulfa with trimethoprime (Table 3). Furthermore, *P. aeruginosa* isolates showed four resistance patterns with a MAR index ranging from 0.44 to 0.66 (Table 4). Interpretation of antibiotic susceptibility to evaluate MDR, XDR, and PDR as in Table 4 showed that 7 out of 14 isolates were multidrug resistant (50%), and five of them were XDR (35.7%).

#### Prevalence of 16SrRNA gene and some antibiotic resistance genes among *Pseudomonas aeruginosa* isolates

**Fig. 1:**
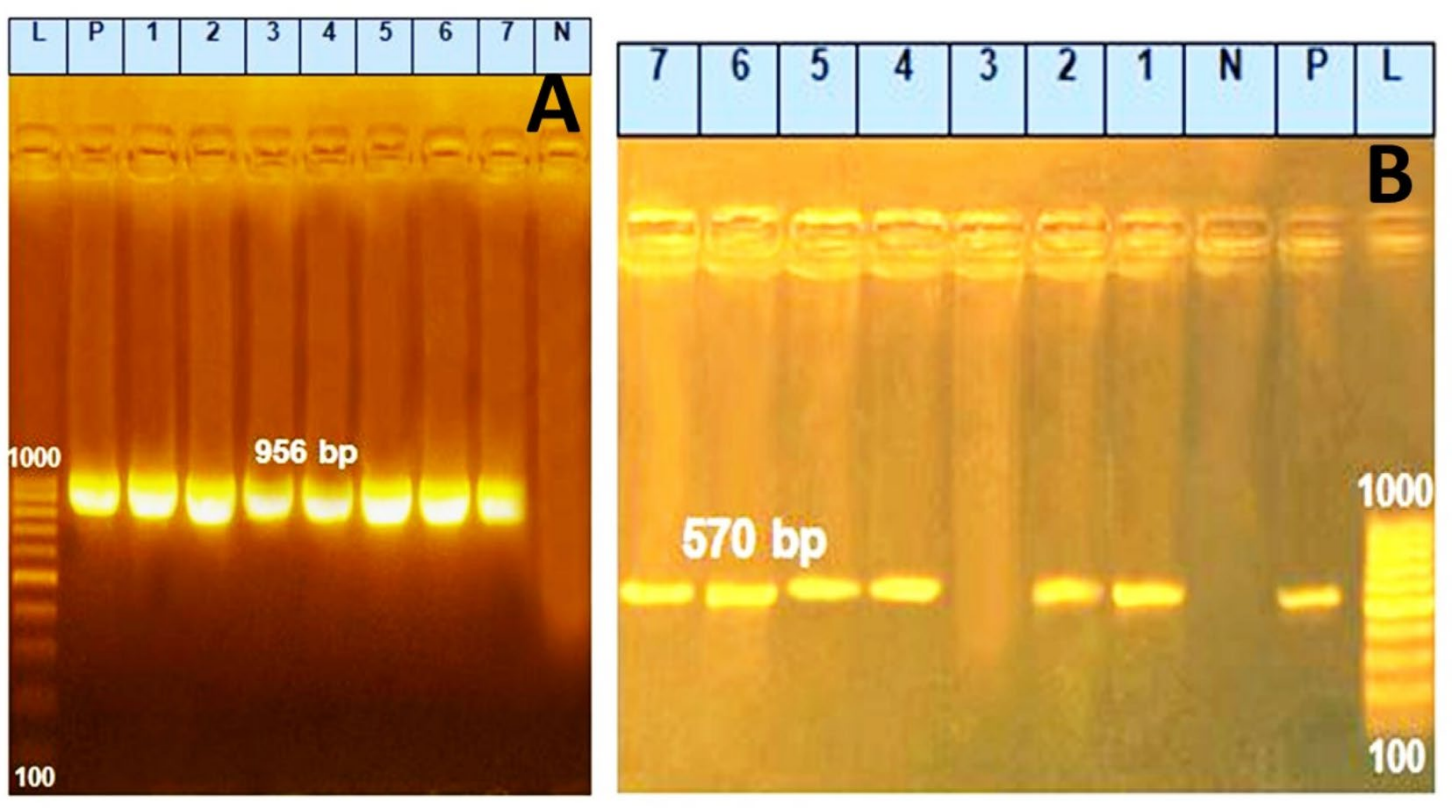
(**A**) Agarose gel electrophoresis of PCR amplification products of *16SrRNA* gene for characterization of *P. aeruginosa*. Lane L: 100–1000 bp molecular size marker. Lane Pos: Control positive *P. aeruginosa 16SrRNA* gene at 956 bp. Lanes 1, 2, 3, 4, 5, 6, 7: Positive *P. aeruginosa* strains *16SrRNA* gene. (**B**) Agarose gel electrophoresis of PCR amplification products of *tetA* gene. Lane L: 100–1000 bp molecular size marker. Lane Pos: Control positive *P. aeruginosa* at 570 bp. Lanes 1, 2, 4, 5, 6, 7: Positive *P. aeruginosa* strains for *tetA* gene.

**Fig. 2:**
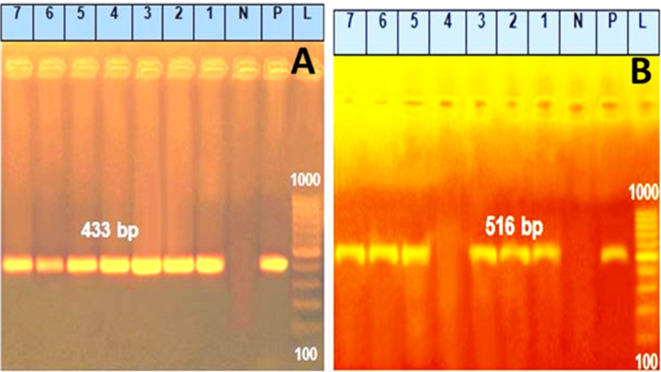
(**A**) Agarose gel electrophoresis of PCR amplification products of *sul1* gene. Lane L: 100–1000 bp molecular size marker. Lane Pos: Control positive *P. aeruginosa sul1* gene at 433 bp. Lanes 1, 2, 3, 4, 5, 6, 7: Positive *P. aeruginosa* strains for *sul1* gene. (**B**) Agarose gel electrophoresis of PCR amplification products of *blaTEM* gene. Lane L: 100–1000 bp molecular size marker. Lane Pos: Control positive *P. aeruginosa blaTEM* gene at 516 bp. Lanes 1, 2, 3, 5, 6, 7: Positive *P. aeruginosa* strains for *blaTEM* gene.

Some of the isolates were screened for harboring the *P. aeruginosa* 16S rRNA gene and some antibiotic resistance genes (*blaTEM, tetA,* and *sul1*)). High prevalence was noted for all genes with a frequency of 100% for 16S rRNA and for *sul1* genes & with a frequency of 85.7% for *blaTEM & tetA genes* as shown in Figs. 1 and 2.

### Second step: results of the in vivo study

#### Clinical signs of experimentally infected fish

**Fig. 3:**
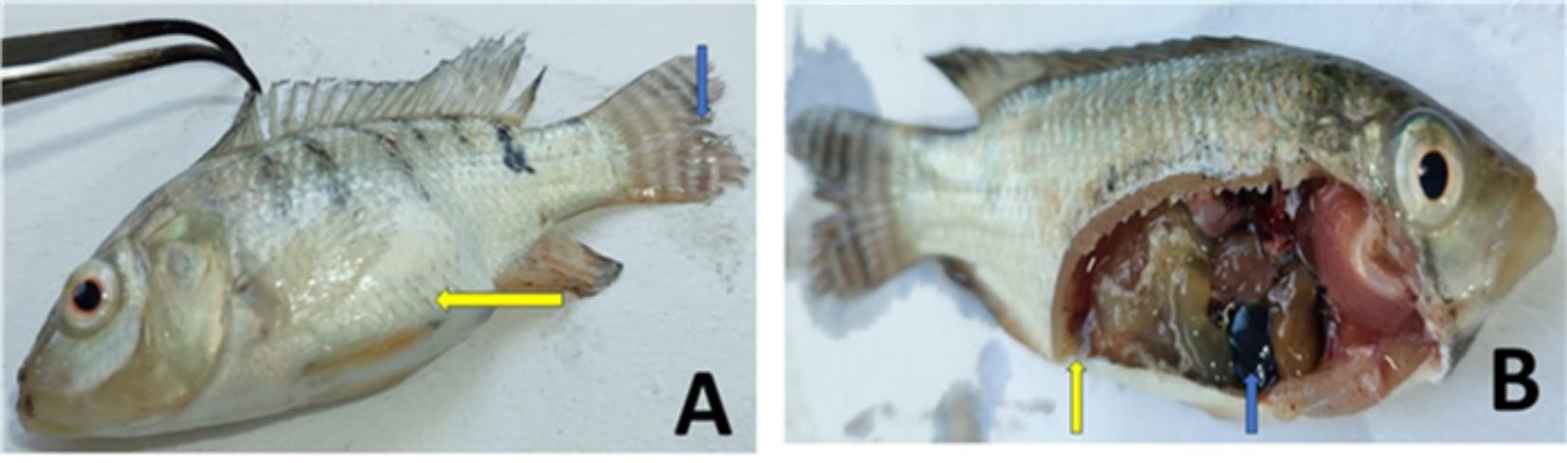
Clinical signs of experimentally infected fish. (**A**) showed enlarged abdomen (yellow arrow) and fine erosion (blue arrow). (**B**) showed ascetic fluid (yellow arrow) and enlarged spleen (blue arrow).

**Fig. 4:**
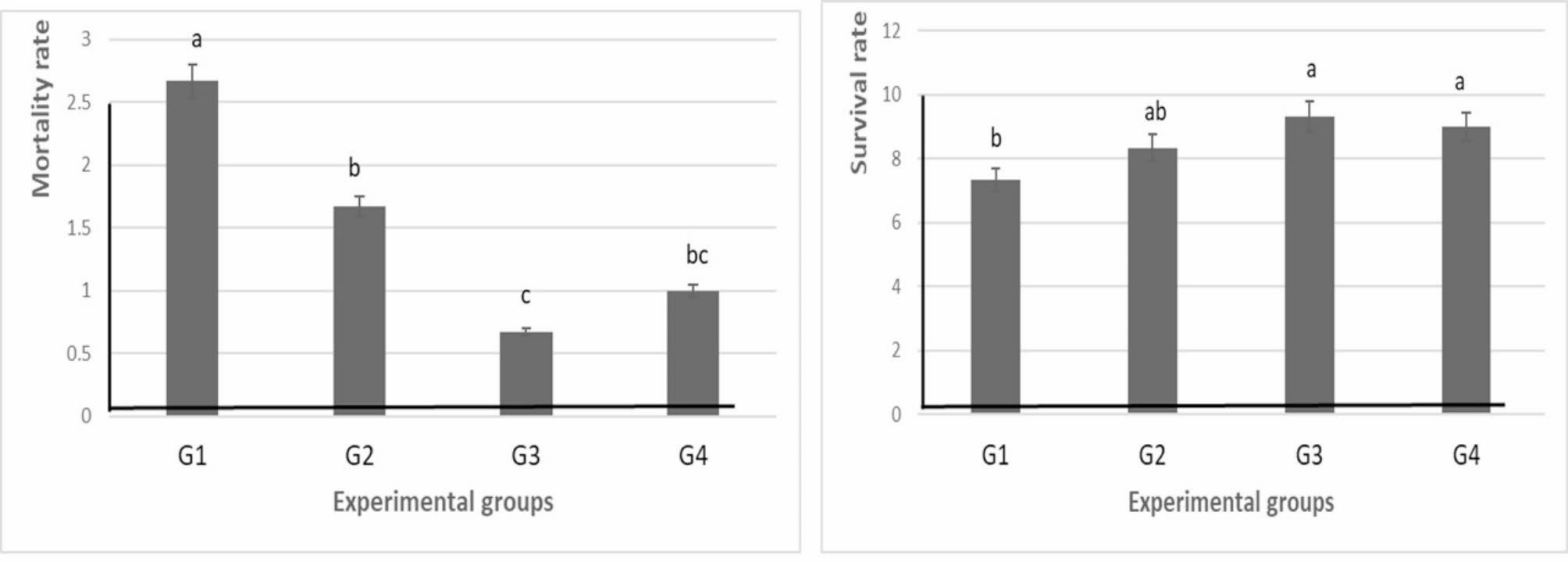
Mortality and survival rates of experimentally infected fish.

Experimentally infected fish showed signs of hemorrhage in the skin at the mouth region, tail and fin rot, corneal opacity, and distended abdomen (Fig. 3). The morbidity rate was decreased in G2, G3, and G4 compared with G1. Death of fish started after 2 days of experimental infection and remained for 7 days (Table 5 and Fig. 4). Moreover, the mortality rate in the control group significantly (*P* ≤ 0.05) increased when compared with the mortality rate of the G2, G3, and G4. Additionally, the survival rate significantly (*P* ≤ 0.05) increased in the G3 and G4 when compared with the control group. Every sick and dead fish was exposed to re-isolation of *P. aeruginosa,* and all gave rise to the growth of *P. aeruginosa* from different organs (spleen, liver, and kidney).

**Table 5:** Mortality of *O. niloticus* experimental groups challenged with *P. aeruginosa.*

Fish group	G1	G2	G3	G4
Total number	30	30	30	30
Dead number	8	5	2	3
Mortality rate	2.67 ± 0.33^a^	1.67 ± 0.33^b^	0.67 ± 0.33^c^	1 ± 0.00^bc^
Survival rate	7.33 ± 0.33^b^	8.33 ± 0.33^ab^	9.33 ± 0.33^a^	9 ± 0.00^a^

#### Growth performance

**Fig. 5:**
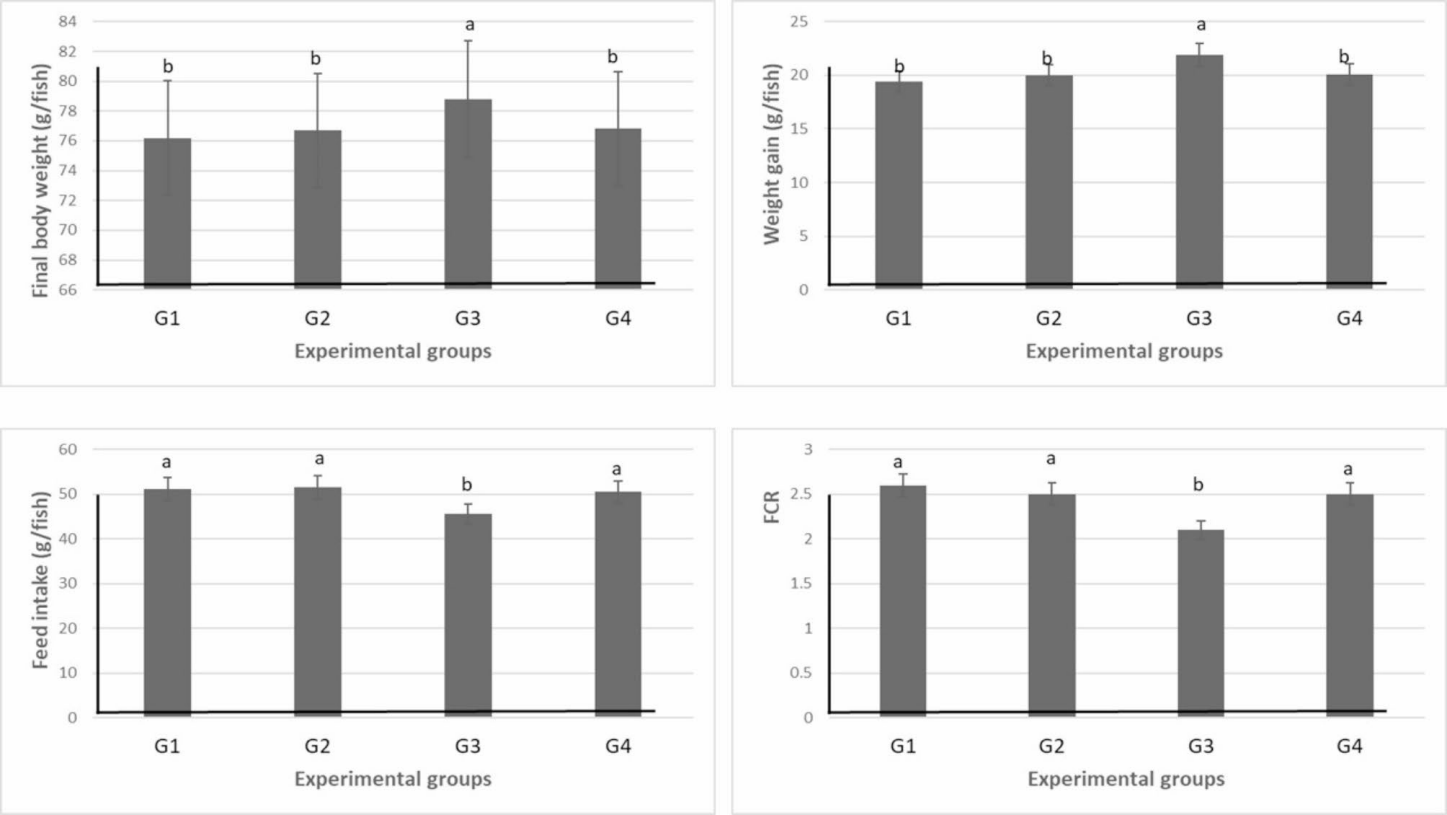
Effect of different doses of levamisole on growth of Nile tilapia.

As indicated in Table 6 and Fig. 5, the inclusion of levamisole at 750 mg/kg diet significantly (*P* ≤ 0.05) increased final body weight and weight gain. However, the FCR improved in the G3 (750 mg/kg diet) with decreased feed intake when compared with other groups.

**Table 6:** Effect of different doses of levamisole on growth of Nile tilapia.

Groups parameters	G1 (0 mg/kg diet)	G2 (500 mg/kg diet)	G3 (750 mg/kg diet)	G4 (1000 mg/kg diet)
Initial body weight (g/fish)	56.8 ± 0.21	56.7 ± 0.06	56.8 ± 0.43	56.7 ± 0.35
Final body weight (g/fish)	76.2 ± 0.26^b^	76.7 ± 0.12^b^	78.8 ± 0.17^a^	76.8 ± 0.38^b^
Weight gain (g/fish)	19.4 ± 0.11^b^	20.0 ± 0.06^b^	21.9 ± 0.44^a^	20.1 ± 0.41^b^
Feed intake (g/fish)	51.2 ± 0.64^a^	51.5 ± 0.05^a^	45.6 ± 0.41^b^	50.5 ± 0.00^a^
FCR	2.6 ± 0.03^a^	2.5 ± 0.01^a^	2.1 ± 0.05^b^	2.5 ± 0.05^a^

Values are means ± standard error. Mean values with different letters at the same row significantly differ at (*P* ≤ 0.05).

#### Immunological assay

**Fig. 6:**
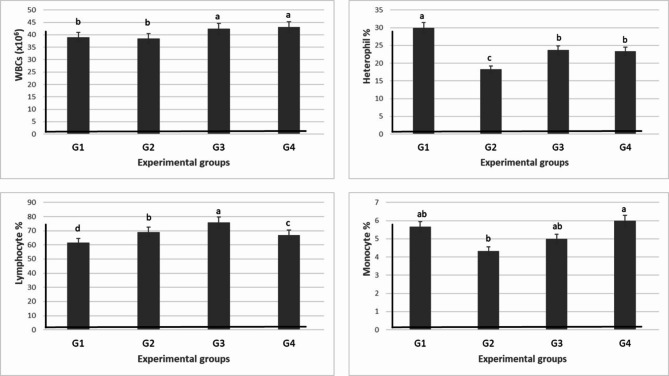
Effect of different doses of levamisole on leukocytic count of Nile tilapia.

Table 7 and Fig. 6 show the results of the immunological assay; the WBCs and lymphocyte count were significantly (*P* ≤ 0.05) increased as a result of levamisole supplementation in G3. On the other side, the high level of levamisole (G4) significantly (*P* ≤ 0.05) increased the WBCs count only in the G4. However, the high level of levamisole significantly increased monocytes, eosinophils, and basophils in the G4 (Table 7).

**Table 7:** Effect of different doses of levamisole on leukocytic count and phagocytic and lysozymes activities, and phagocytic index of Nile tilapia.

Groups parameters	G1 (0 mg/kg diet)	G2 (500 mg/kg diet)	G3 (750 mg/kg diet)	G4 (1000 mg/kg diet)
WBCs (× 10^6^)	39.08 ± 0.49^b^	38.54 ± 0.26^b^	42.42 ± 0.77^a^	43.11 ± 0.45^a^
Heterophil %	30 ± 0.58^a^	18.33 ± 0.88^c^	23.67 ± 0.88^b^	23.33 ± 0.33^b^
Lymphocyte %	61.43 ± 0.69^d^	69.15 ± 0.67^b^	75.84 ± 0.03^a^	67.07 ± 0.34^c^
Monocyte %	5.67 ± 0.33^ab^	4.33 ± 0.33^b^	5 ± 0.57^ab^	6 ± 0.58^a^
Eosinophils %	2.67 ± 0.33^ab^	1.33 ± 0.33^c^	2 ± 0.0^bc^	3.33 ± 0.33^a^
Basophils %	0.24 ± 0.02^ab^	0.16 ± 0.03^c^	0.19 ± 0.01^bc^	0.26 ± 0.02^a^
Phagocytic activity %	19.33 ± 0.47^c^	23.80 ± 0.74^b^	28.31 ± 0.22^a^	20.49 ± 0.18^c^
Phagocytic index	3.69 ± 0.22^b^	4.46 ± 0.40^b^	5.44 ± 0.37^a^	4.04 ± 0.10^b^
Lysozymes activity	33.08 ± 0.26^c^	40.52 ± 0.71^b^	44.91 ± 0.41^a^	39.55 ± 0.80^b^

Values are means ± standard error. Mean values with different letters at the same row significantly differ at (*P* ≤ 0.05).

**Fig. 7:**
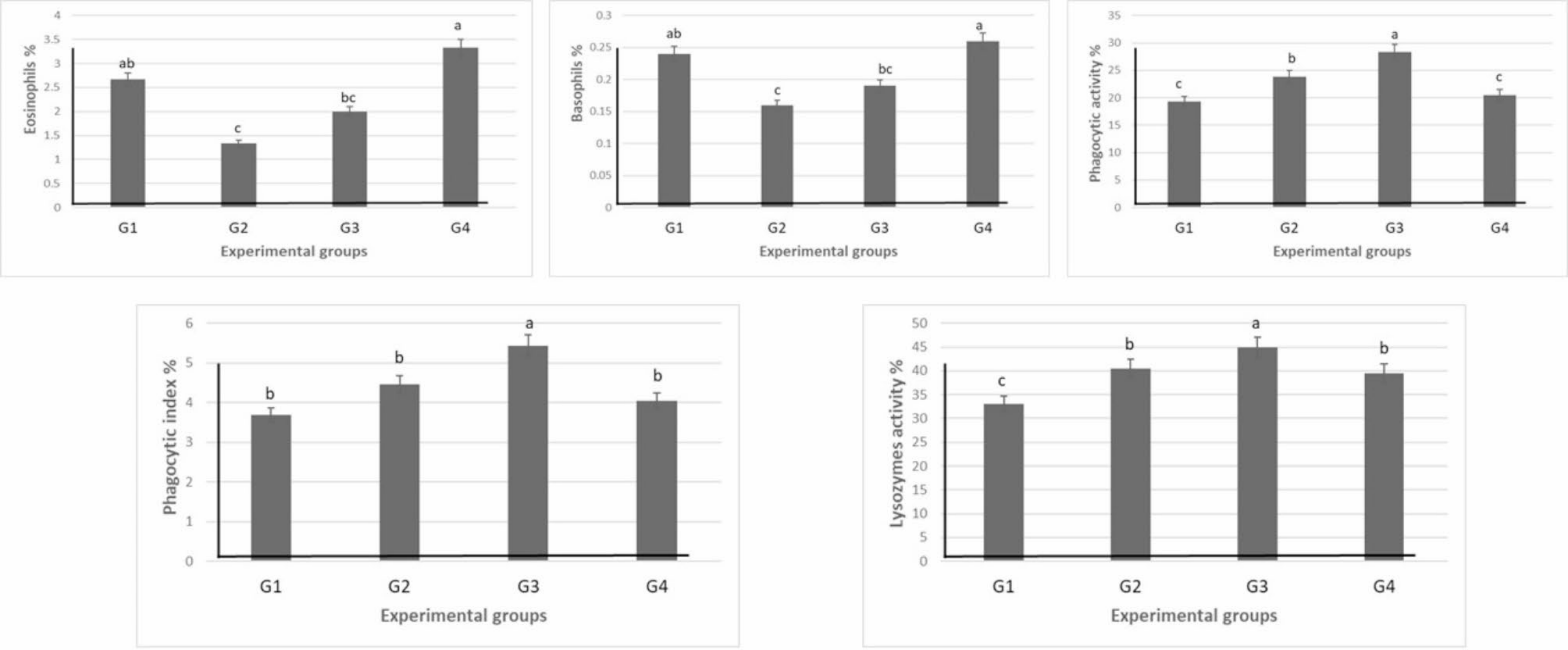
Effect of different doses of levamisole on phagocytic and lysozymes activities, and phagocytic index of Nile tilapia.

The use of levamisole significantly (*P* ≤ 0.05) increased the activity of phagocytic and lysozyme in the G3. Meanwhile, the activity and index of the three different measurements were significantly (*P* ≤ 0.05) decreased with the high dose of levamisole (G4) (Table 7 and Fig. 7).

#### Safety of levamisole supplementation

**Fig. 8:**
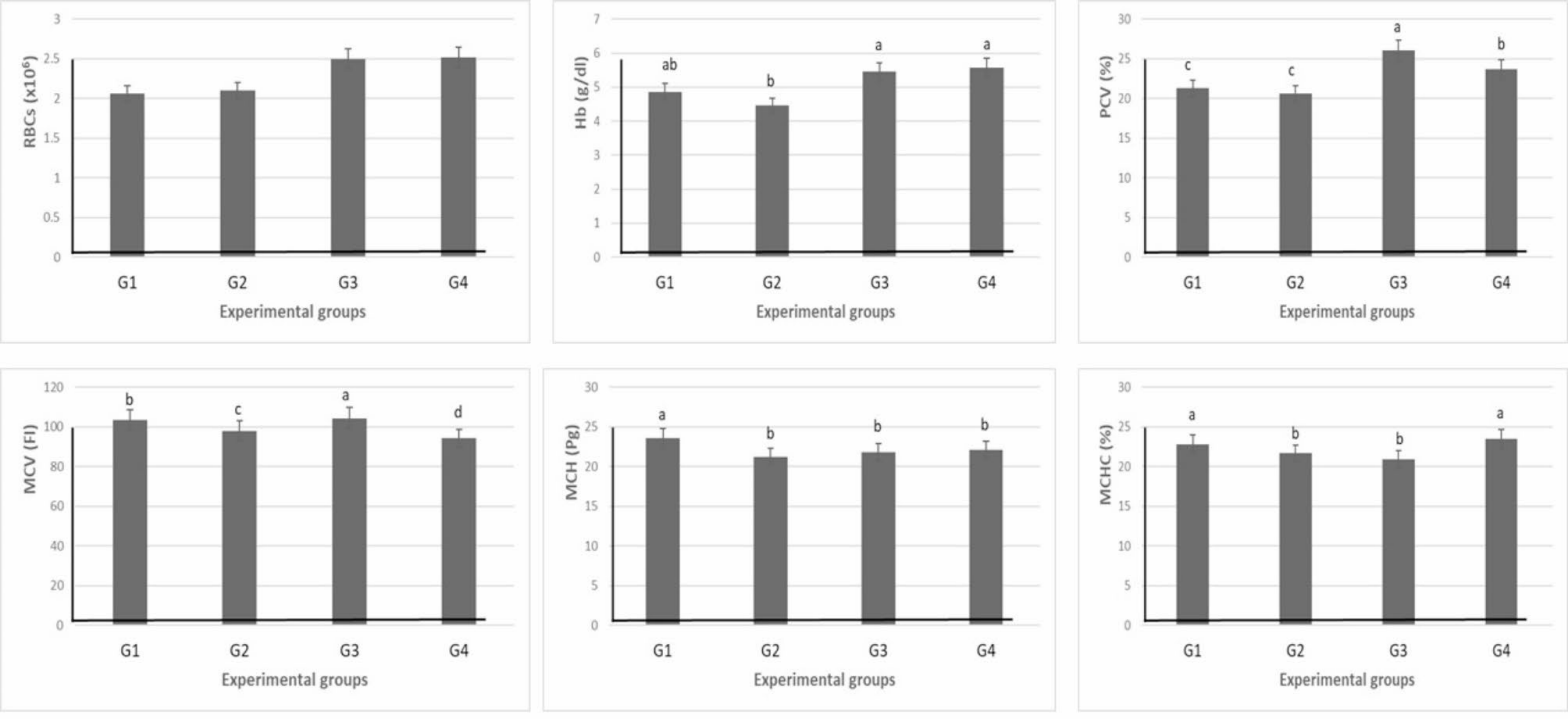
Effect of different doses of levamisole on hematology of Nile tilapia.

The safety of levamisole supplementation was assessed through measurements of hematological parameters and liver function tests. Fish fed different doses of levamisole had no effect on the RBCs count but significantly (*P* ≤ 0.05) increased Hb with high doses (750 and 1000 mg/kg diet) compared with the fish fed on the basal diet and low dose of levamisole. Moreover, PCV and MCV significantly (*P* ≤ 0.05) increased in the G3 (750 mg/kg diet) when compared with the other groups. MCH was higher in the G1 when compared with the other groups. Meanwhile, MCHC was higher in G1 and G4 when compared with the other groups (Table 8 and Fig. 8).

**Table 8:** Effect of different doses of levamisole on hematology of Nile tilapia.

Groups parameters	G1 (0 mg/kg diet)	G2 (500 mg/kg diet)	G3 (750 mg/kg diet)	G4 (1000 mg/kg diet)
RBCs (× 10^6^)	2.06 ± 0.09	2.10 ± 0.12	2.50 ± 0.18	2.52 ± 0.17
Hb g/dl	4.86 ± 0.11^ab^	4.46 ± 0.21^b^	5.45 ± 0.35^a^	5.57 ± 0.28^a^
PCV %	21.27 ± 0.82^c^	20.59 ± 0.92^c^	26.09 ± 0.38^a^	23.7 ± 0.27^b^
MCV Fl	103.34 ± 0.15^b^	98.03 ± -.27^c^	104.48 ± 0.37^a^	94.19 ± 0.46^d^
MCH Pg	23.62 ± 0.49^a^	21.25 ± 0.22^b^	21.85 ± 0.59^b^	22.13 ± 0.42^b^
MCHC %	22.85 ± 0.37^a^	21.67 ± 0.21^b^	20.92 ± 0.33^b^	23.50 ± 0.07^a^

Values are means ± standard error. Mean values with different letters at the same row significantly differ at (*P* ≤ 0.05).

**Fig. 9:**
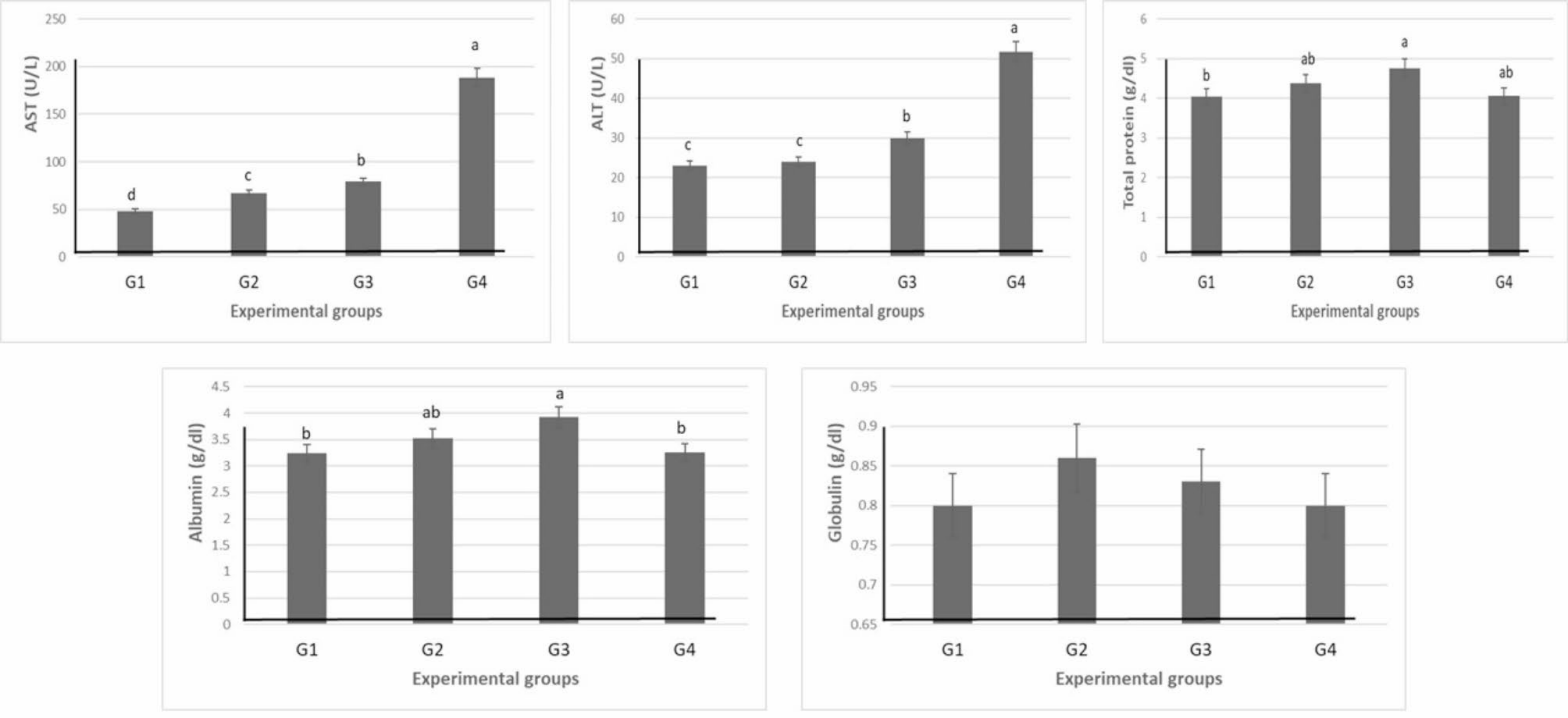
Effect of different doses of levamisole on liver function test of Nile tilapia.

The use of levamisole significantly (*P* ≤ 0.05) increased the activity of AST in the levamisole different treatment in a dose-dependent manner. Meanwhile, the ALT was significantly (*P* ≤ 0.05) increased with the high doses only (G3 and G4). However, the different levels of levamisole significantly increased the serum proteins (total protein and albumin) in G3 and G4 without affecting the globulin (Table 9 and Fig. 9).

**Table 9:** Effect of different doses of levamisole on liver function test of Nile tilapia.

Groups parameters	G1 (0 mg/kg diet)	G2 (500 mg/kg diet)	G3 (750 mg/kg diet)	G4 (1000 mg/kg diet)
AST (U/L)	48.33 ± 0.88^d^	67.33 ± 0.33^c^	79 ± 0.57^b^	188.67 ± 0.33^a^
ALT (U/L)	23 ± 0.58^c^	24 ± 0.58^c^	30 ± 0.58^b^	51.67 ± 0.88^a^
Total protein (g/dl)	4.04 ± 0.10^b^	4.38 ± 0.23^ab^	4.75 ± 0.33^a^	4.06 ± 0.06^ab^
Albumin (g/dl)	3.24 ± 0.09^b^	3.52 ± 0.23^ab^	3.92 ± 0.19^a^	3.26 ± 0.07^b^
Globulin (g/dl)	0.8 ± 0.03	0.86 ± 0.01	0.83 ± 0.15	0.80 ± 0.04

Values are means ± standard error. Mean values with different letters at the same row significantly differ at (*P* ≤ 0.05).

## Discussion

*Pseudomonas* species identification is necessary for precise diagnosis, epidemic forecasting, and the application of prophylactic and/or preventive interventions in aquaculture^37^. Since *P. aeruginosa* causes illnesses linked to healthcare for many customers, serious efforts to detect it further were taken into consideration due to its importance to public health as well as its economic impact.

The results of gross and PM lesions of the collected fish in the first step are similar to those reported by Magdy et al.^38^, Abd El Tawab et al.^39^, Algammal et al.^26^, and Yaseen et al.^40^; these clinical and postmortem findings were typical of *Pseudomonas* septicemia. All the retrieved isolates exhibited the typical phenotypic characteristics, culture characters, and biochemical characteristics of *P. aeruginosa*. This is following those reported by Abd El Tawab et al.^39^, Algammal et al.^26^, and Mohamed et al.^5^.

Moreover, the presentation of *P. aeruginosa* in the Nile tilapia samples is nearly similar to Osman et al.^1^ and Abou Elez et al.^41^, who found that *P. aeruginosa* was recovered in 12% and 15.6% of the samples. Lower results were reported in Uganda with 5.1% by Wamala et al.^42^ and 5% by Shahrokhi et al.^4^ from fresh fish in Iran, and by Mumbo et al.^43^ with 4.4% from Nile tilapia in Kenya. In *O. niloticus*, a higher prevalence was observed by Magdy et al.^38^, Algammal et al.^26^, and Mohamed et al.^5^ with an incidence rate of 34.4%, 32.72%, and 29%, respectively. Geographical distribution, climatic conditions, host vulnerability, and sample collection season may all have an impact on prevalence variations.

Antibiotic sensitivity testing should be used on a regular basis in order to choose the most effective antibiotic and solve this issue. The results of susceptibility of all *P. aeruginosa* isolates to 9 antimicrobial agents align with those of Mohamed et al.^5^, who asserted that all isolates of *P. aeruginosa* showed a 100% sensitivity rate to amikacin and ciprofloxacin. Also, with Abd El Tawab et al.^39^, Eid et al.^44^, and Ali et al.^45^. Therefore, we advise using amikacin, norfloxacin, and ciprofloxacin to treat *P. aeruginosa* infections. Conversely, isolates of *P. aeruginosa* were said to be resistant to ciprofloxacin by Benie et al.^46^. Approximately 50% of the isolates were multidrug resistant to five to six medicines. This is consistent with Algammal et al.^26^, who found that 50 strains (50/90, 55.5%) exhibited multi-drug resistance to four antimicrobial agents: amoxicillin, cefotaxime, tetracycline, and gentamicin. On the other hand, according to Mohamed et al.^5^, all isolates of *P. aeruginosa* were multiple antimicrobial resistant (MAR). The multiple antimicrobial resistance (MAR) index is nearly inconsistent with Mohamed et al.^5^ and Darwish et al.^47^. According to Magiorakos et al.^29^, multiple drug resistance (MDR) is defined as resistance to at least one agent in three or more antimicrobial categories. Moreover, extensive drug resistance (XDR) is defined as resistance to at least one agent in all antimicrobial categories except two or one category (i.e. bacterial isolates remain susceptible to only one or two categories). Pan drug resistance (PDR) is defined as resistance to all agents in all antimicrobial categories. The numbers of the isolates of XDR and MDR nearly similar results were detected by Abou Elez et al.^41^, who exhibited MDR and XDR, 61.5% and 23.1%, respectively. Additionally, PDR *P. aeruginosa* was not detected, which is inconsistent with Abd El-Baky et al.^48^ and Abou Elez et al.^41^.

The molecular-based identification of this pathogen could overcome the drawbacks of traditional techniques and provide a comprehensive understanding of the ecological significance of such infections^49^. The result of the molecular-based identification is in accordance with Algammal et al.^26^, who revealed that every isolate tested positive for the species-specific *16S rRNA* gene by PCR. *P. aeruginosa* may develop innate and/or acquired resistance to multiple antimicrobial agents^50^ as a result of the active efflux of antibiotics and the permeability of its outer membrane^8^. To prevent the formation of antibiotic-resistant strains that pose a threat to world health, molecular typing of the majority of inherited antibiotic-resistance genes should be carried out.

*Pseudomonas aeruginosa’s* resistance to first-, second-, and third-generation penicillin and other β-lactam antibiotics is mostly caused by Extended Spectrum Beta-lactamases (ESBLs). According to Peymani et al.^51^, the primary genes for Extended Spectrum β-lactamases that cause this kind of resistance are *blaCTX-M* and *blaTEM*. Numerous types of sulfonamide and tetracycline antibiotics are found in aquaculture as a result of their widespread use in treating bacterial and protozoan illnesses^52^. As a result, sulfonamide and tetracycline antibiotic resistance have been a common issue in aquaculture operations^53^. Due to its extensive use, rapid rate of excretion, high solubility, and environmental persistence, sulfonamide merits particular consideration^54^. Sulfonamide-resistant bacteria can persist in the aqueous environment for five or 10 years even in the absence of selective pressure. It has been confirmed that the bacteria resistant to sulfonamide are more persistent than the sulfonamide itself^55^. The examined strains were 100%, 85.7%, and 857% positive for* sul1*, *tetA,* and *blaTEM* genes; these results are nearly compatible with those of Algammal et al.^26^ and Mumbo et al.^43^.

The two main issues in aquaculture are fish growth and disease resistance. One of the most well-known immunostimulants for aquaculture that promotes growth is levamisole^56^. The experimental challenge of fish with *P. aeruginosa* in the present study showed some clinical signs and PM lesions. These signs and lesions are matched with those obtained by Algammal et al.^26^. Additionally, the result of the mortality percentage is inconsistent with Maqsood et al.^57^, who reported that the experimental groups that received varying doses of levamisole had a lower mortality rate. Moreover, the result of the survival rate is compatible with Bedasso^58^.

Regarding the results of the body weight, consistent with the current investigation, levamisole use has been shown by Alishahi et al.^59^ to accelerate the growth rate of Oscar fish (*Astronotus ocellatus*). Furthermore, higher weight gain may result from using levamisole in *Clarias fuscus*^56^. Levamisole use greatly enhanced haematological indices and growth performance^60^. Li et al.^61^ found that low levels of dietary levamisole in hybrid striped bass significantly improve growth, immunity, and resistance against experimental infection with *Streptococcus iniae* and *Aeromonas hydrophila*. However, the potential impact of levamisole overdose on immunity and disease resistance was not shown. The current investigation also supported the earlier finding that excessive use of levamisole (1000 mg/kg) led to growth depression and decreased feed efficiency^61,62^.

On the other hand, the results of the hematology and leukogram are compatible with Biller-Takahashi et al.^63^, who reported that administering levamisole raised the hematocrit, serum bactericidal activity, and antibody titer in addition to the quantity of thrombocytes, leukocytes, and red blood cells in the pacu. Eslami and Bahrekazemi^64^ found that levamisole increased the hemoglobin and hematocrit of beluga. Maqsood et al.^57^ found that adding levamisole to the feed of common carp fingerlings undoubtedly improves their non-specific immunity, boosts their resistance to infection, lowers fish mortality, and promotes fish growth. In contrast to our results, Eslami and Bahrekazemi^64^ reported that levamisole did not affect the leukocytic count of beluga but increased lysosomal activity. Moreover, levamisole does not affect the hematology of catfish and insignificantly increases the total leukocytic count^65^. However, this difference may be attributed to the dose of levamisole, the type of fish, and the route of administration of the levamisole.

Levamisole can be delivered by injection, oral consumption, or immersion, and has immunostimulatory effects when taken orally^24^. The least amount of stress is caused by oral administration; nevertheless, the drug’s effectiveness may be diminished by gastrointestinal enzymes, and its impact may change depending on feed intake^66^. Consequently, it is crucial to confirm the safety and effectiveness of any administration technique^67^. Moreover, an essential metric for evaluating the well-being of farmed fish and verifying safety following medication treatment is biochemical analysis^68^. Of the many characteristics, the two vital blood enzymes that are most closely linked to liver function are AST and ALT^69^. When liver function is considered normal, these enzymes stay within a certain range; however, when liver cell injury occurs, they leak into the blood and become more concentrated^70^. Results of the liver function are compatible with the results of Maqsood et al.^57^, who found that adding levamisole to the feed of common carp fingerlings elevates serum proteins. On the contrary, Eslami and Bahrekazemi^64^ reported that levamisole does not affect the serum proteins or liver enzyme activity of beluga. Moreover, Biller-Takahashi et al.^63^ found that lysozyme activity and the level of total protein, albumin, and globulin were not affected by dietary levamisole. Aly et al.^65^ reported that levamisole does not affect serum proteins. Woo et al.^71^ showed that levamisole significantly increased the activity of AST and ALT in *Sebastes schlegelii*. However, Ulaiwi^72^ reported the protective effect of levamisole against broiler aflatoxicosis through decreased ALT and AST activities. However, Woo et al.^71^ reported that the most suitable and safe method for levamisole HCl administration to Korean rockfish is oral.

## Conclusion

Our results of the first step showed that *P. aeruginosa* was present in 14% of Nile tilapia overall, with half of the isolates being multidrug-resistant to 5–6 medications. Furthermore, the high frequency of antibiotic resistance genes suggests that strong regulation of the overuse and needless use of antibiotics is imperative and urgent since there is a considerable risk of antibiotic resistance genes spreading to the microbial population and posing a risk to human health. Thus, frequent fish farm monitoring is essential for detecting *P. aeruginosa* at a wide spectrum, which is required for pathogen identification and aquaculture disease outbreak control. The recovery of MDR and XDR strains of *P. aeruginosa* indicates improper use of antibiotics. Furthermore, it is important to promote the use of alternate, non-antibiotic management strategies for bacterial infections in farmed fish. Moreover, from the results of the second step, we can conclude that levamisole increased the Nile tilapia’s growth and resistance to bacterial infection. Furthermore, it was asserted that 750 mg of levamisole per kilogram of diet is the ideal dosage for fish in a practical diet under the present experimental setting. So, emphasis on the usage of levamisole as a non-antibiotic alternative control method for bacterial infections in farmed fish is valuable method.

## Data Availability

The datasets used and/or analyzed during the current study available from the corresponding author on reasonable request.
